# Impact of historic histopathologic sample review on the risk of recurrence in patients with differentiated thyroid cancer

**DOI:** 10.20945/2359-3997000000020

**Published:** 2018-03-23

**Authors:** Fabián Pitoia, Fernando Jerkovich, Carolina Urciuoli, Florencia Falcón, Andrea Páes de Lima

**Affiliations:** 1 Universidad de Buenos Aires University of Buenos Aires Division of Endocrinology Buenos Aires Argentina Division of Endocrinology, University of Buenos Aires, Buenos Aires, Argentina; 2 Universidad de Buenos Aires University of Buenos Aires Department of Pathology Buenos Aires Argentina Department of Pathology, University of Buenos Aires, Buenos Aires, Argentina

**Keywords:** Risk of recurrence, response to therapy, differentiated thyroid cancer, historic histopathologic review

## Abstract

**Objective:**

To compare the historic risk of recurrence (RR) and response to therapy to risk stratification estimated with historical pathology reports (HPRs) and contemporary re-review of the pathological slides in patients with differentiated thyroid cancer (DTC).

**Subjects and methods:**

Out of 210 DTC patients with low and intermediate RR who underwent total thyroidectomy and remnant ablation in our hospital, 63 available historic pathologic samples (HPS) were reviewed. The RR and the response to therapy were evaluated considering historical histological features (histological type, tumor size, capsular invasion, number of lymph node metastases) and then, reassessed after observing additional histological features (vascular invasion, extrathyroidal extension, size of lymph node metastases, presence of extranodal extension, and/or status of the resection margins).

**Results:**

A change in the RR category was observed in 16 of 63 cases (25.4%). Out of 46 patients initially classified as low RR, 2 patients were reclassified as intermediate RR, 4 as high RR, and 1 as noninvasive follicular thyroid neoplasm with papillary-like nuclear features (NIFTP). Out of 17 patients initially classified as intermediate RR, 3 were reassigned to the low RR group, 5 as high RR, and 1 as NIFTP. The percentages of structural incomplete response at final follow-up changed from 2.2 to 0% (*p* = 1) in patients with low RR and from 6.3 to 20% (*p* = 0.53) in patients with intermediate RR.

**Conclusion:**

A detailed report of specific features in the HPR of patients with DTC might give a more accurate RR classification and a better estimation of the response to treatment.

## INTRODUCTION

The pathological examination of thyroid samples is important for establishing the diagnosis of differentiated thyroid carcinoma (DTC), and it also provides useful information for the risk stratification assessment (1). On the other side, the TNM AJCC/UICC staging system is recommended for predicting disease mortality (1).

The 2009 version of the American Thyroid Association (ATA) thyroid cancer guidelines proposed a three-tiered risk stratification system in which specific clinical-pathologic features are used to estimate the risk of structural incomplete response (SIR) and the probability of an excellent response to treatment (2). The variables taken into account for risk stratification in the 2009 version of the ATA guidelines were: evidence of microscopic or gross extrathyroidal extension, presence of aggressive histology, completeness of tumor resection, existence of metastatic lymph node/s and/or distant metastasis, and observation of RAI-avid disease outside the thyroid bed (2).

In this first ATA risk of recurrence (RR) classification system, many important features were not taken into account. For example, any patient with lymph node metastasis was deemed to have an intermediate RR. In fact, the risk of structural disease can vary from 4% if having fewer than five metastatic lymph nodes, or 5% if all involved lymph nodes are less than 0.2 cm, to 19% if more than five lymph nodes are involved or 27%-32% if any metastasis in the lymph node is larger than 3 cm (3,4). Additionally, follicular thyroid carcinomas (FTCs) with vascular invasion, initially classified by the 2009 ATA system as intermediate RR, have an excellent prognosis when minor vascular invasion is present but may have a high RR (30%-55%) when more than four foci are demonstrated in the pathological examination (5,6).

Although the 2009 risk stratification system has proven to be a valuable tool for initial risk stratification in DTC (7–10), modifications were required to better assess the RR of DTC patients. The recently published 2015 version of the ATA guidelines included specific prognostic variables: the extent of vascular invasion, the extent of lymph node involvement (number and size of metastatic lymph nodes and presence of extranodal extension), and mutational status (*BRAF* and/or *TERT* when available) (1). As a result, the low risk category was expanded to include patients with small-volume lymph node metastases (clinical N0 or < 5 pathologic N1 micrometastases, < 0.2 cm in the largest dimension), the intermediate RR group now considers only a subset of patients with lymph node metastases (clinical N1 or > 5 pathologic N1 with all involved lymph nodes < 3 cm in the largest dimension), and a second group, with intermediate to high risk of recurrence, now includes those cases with large-volume lymph node involvement (any metastatic lymph node > 3 cm in the largest dimension, > 3 lymph node metastasis with extranodal extension) and FTC with extensive vascular invasion (> 4 foci of vascular invasion or extracapsular vascular invasion). The 2015 guidelines defined this new version of the RR as the “modified risk stratification system from ATA 2009 guidelines” (MRSS ATA 2009) and were validated by our and other groups around the world in ablated and non-ablated patients (11–14).

The vascular invasion (≥ or < 4 foci), the magnitude of extrathyroidal extension, the size of the largest metastatic focus in lymph nodes, the presence of extranodal extension, and the status of the resection margins were not uniformly informed in the historic pathologic reports that were used to assess the RR of DTC patients in our hospital and most centers in Latin America, with the exception of the number of lymph node metastases.

However, the additional benefit of adding the previously mentioned specific features for reclassifying patients has not yet been established. Therefore, our objective was to compare the historic RR and the responses to treatment to risk stratification systems estimated with historical pathology reports and contemporary re-review of the slides by two expert pathologists.

## SUBJECTS AND METHODS

### Inclusion criteria

The file records from our database of 610 patients with DTC were reviewed for the period January 2001 to March 2016. The inclusion criteria were i) to be 18 years old or older, ii) to have had a follow-up for at least 3 years, iii) to have received a total thyroidectomy with or without lymph node dissection and remnant ablation with radioiodine after thyroid hormone withdrawal (THW) or recombinant human thyrotropin (rhTSH). Lymph node dissection was performed when clinical pathological lymph nodes were diagnosed previous to surgery, when the surgeons had a suspicion of lymph node metastasis, or when the size of the thyroid tumor was greater than 4 cm in diameter.

Out of 268 patients who met these inclusion criteria, 210 patients with low and intermediate RR were considered for analysis. The RR was assessed as described in Table 1. Because the historic pathological reports did not systematically detail the size of the lymph node metastasis, the extent of extranodal extension, the presence of vascular invasion, or the microscopic invasion of surgical margins, these variables were not taken into account for the initial RR stratification of these patients. The number of metastatic lymph nodes (> 5, intermediate RR, and < 5 low RR), as it was available in the pathological report, was considered for the initial RR classification. Also, as it was validated in a previous analysis from our cohort of DTC patients in our institution, intrathyroidal tumors with minor extrathyroidal extension (T3) were considered in the low RR group (11). One hundred fifteen patients were excluded because they had been operated on in other institutions and the pathological slides were not available in our hospital. Also, 32 patients were excluded due to insufficient histopathological material.

**Table 1:** Risk of recurrence classification considering specific histological features

Low RR	Classical and follicular variant PTC Minor extrathyroidal extension < 4 foci of vascular invasion < 5 LN metastasis 0.2-3 cm All LN metastasis < 0.2 cm No evidence of extranodal extension
Intermediate RR	Aggressive histology (tall cell, columnar cell, Hürthle cell carcinomas, oncocytic PTC) > 5 LN metastasis 0.2-3 cm Extranodal extension in < 3 LN metastasis
Intermediate-high RR	≥ 4 foci of vascular invasion Extranodal extension in > 3 LN metastasis Any LN > 3 cm Surgical margins involved

RR: risk of recurrence; PTC: papillary thyroid cancer; LN: lymph node.

Sixty-three low and intermediate RR patients were included in the final analysis. The historic histopathologic samples (HPS) of these 63 patients were reviewed by two senior pathologists from our hospital (P.L.A. and F.F.). The analysis consisted of a first diagnosis provided by F.F. and a re-review and agreement with P.L.A., to give a final homogeneous result.

### Pathological analysis

The RR was reassessed considering the following variables: histologic variant, number of foci of vascular invasion, volume of lymph node metastases (number of metastatic lymph nodes and size), presence of extranodal extension, and status of the resection margins. The RR classification according to these new histological features can be seen in Table 1.

Patients having an encapsulated follicular variant of papillary thyroid carcinoma (FVPTC) were reclassified as noninvasive follicular thyroid neoplasm with papillarylike nuclear features (NIFTP), currently considered as a benign neoplasm according to the criteria proposed by Nikoforov and cols. (15).

### Ablation protocol

Patients received radioiodine ablation after stimulation with rhTSH (*n* = 31) or endogenous TSH (*n* = 32), using activities of 3.70 GBq (100 mCi 131-I) for low risk (ATA 2009 RR, Table 1) and 5.55 GBq (150 mCi 131-I) for intermediate risk (ATA 2009 RR, Table 1). Endogenous TSH stimulation was obtained after thyroid hormone withdrawal for at least 3 weeks. Radioiodine was administered following that interval, in all cases with TSH levels above 50 mUI/L.

### Clinical endpoints

The initial response to therapy, defined as the best response to initial therapy assessed at the 12 ± 5 months visit, and the clinical status at final follow-up were evaluated according to the response-to-therapy classification proposed by the ATA 2015 guidelines (2). The initial and final responses were compared to the risk stratification before and after reviewing the pathological samples.

### Statistical analysis

Quantitative variables were expressed as the mean ± standard error mean, median, and range, while qualitative variables were shown as percentages. To evaluate significant differences in the percentages of the responses-to-therapy before and after the pathologic review, we analyzed two-way contingency tables by the Fisher exact test or 2 x 2 contingency table by the chisquare test when appropriate. Statistical analysis was developed using the SPSS statistical software (SPSS version 20.0, Inc., Chicago, IL, USA). We considered *p* < 0.05 to be statistically significant for all the analyses.

## RESULTS

The demographics, clinical features, and MRSS ATA 2009 for each of the 63 patients included in this study are seen in Table 2.

**Table 2:** Baseline characteristics of 63 patients with DTC included in the study

Gender		
	Female/Male	58 (92%)/5 (8%)
Age (years)		
	Mean ± SEM	48.3 ± 16.1
	Median (range)	47 (21.2-76)
Histology		
	PTC classic variant	47 (74.6%)
	PTC follicular variant	13 (20.6%)
	PTC oncocytic variant	2 (3.1%)
	PTC tall cell variant	1 (1.7%)
Cervical lymphadenectomy		
	No	18 (28.5%)
	Central	40 (63.4%)
	Central and lateral	5 (9.1%)
Bilaterality, multifocality, and capsular invasion		
	Bilateral	18 (28.5%)
	Multifocal	13 (20.6%)
	Capsular invasion	16 (25.3%)
TNM stage		
	I	45 (71.4%)
	II	1 (1.7%)
	III	17 (26.9%)
MRSS ATA 2009 before HSR		
	Low	46 (73%)
	Intermediate	17 (27%)
^131^I Cumulative Dose (mCi)		
	Mean ± SEM	230 ± 116
	Median (range)	150 (100-350)

MRSS ATA 2009: modified risk stratification system from ATA 2009 guidelines; SEM: standard error mean.

The percentages of new specific histological features assessed after the pathological review are detailed in Table 3.

**Table 3:** Characteristics of initial pathological reports compared with those obtained after histopathological sample review

Variable		Informed in initial report	Informed after HSR
Number of foci of vascular invasion (> 4)		Not informed	0/63 (0%)
Surgical margins involved		Not informed	7/63 (11.1%)
Metastatic lymph node size		Not informed	
	< 0.2 cm		6/19 (31.6%)
	0.2-1 cm		5/19 (26.3%)
	> 1 cm < 3 cm		5/19 (26.3%)
	> 3 cm		3/19 (15.8%)
Extranodal extension		Not informed	3/19 (15.7%)
	> 3 lymph nodes		1/19 (5.2%)
Multifocality		18 (28.5%)	18 (28.5%)
Bilaterality		13 (20.6%)	13 (20.6%)
Necrosis		Not informed	0/63 (0%)
Extrathyroidal extension		16/63 (25.6%)	
	Minimal	Not informed	24/63 (38%)
	Gross (> 1 cm)	Not informed	7/63 (11.1%)

HSR: histopathological sample review.

After the review of the pathologic slides, a change in the RR category was made in 25.4% (16/63) of the cases. Out of 46 patients initially classified as low RR, 2 patients were reclassified as intermediate RR because of extranodal extension in fewer than 3 lymph nodes, 4 patients were reassigned to the intermediate-high RR group because of surgical margins involved, and 1 patient finally was diagnosed as harboring an NIFTP (Table 4).

**Table 4:** Reasons for reclassification of patients after the historic pathology slide review

MRSS ATA 2009	MRSS ATA 2009 after reviewing the HPS	Reason for changing the RR
Low RR (*n* = 46)	Low RR (*n* = 39)	
	Intermediate RR (*n* = 2)	*n* = 1, PTC oncocytic variant (previously classic variant PTC)
		*n* = 1, extranodal extension in < 3 LN metastasis
	Intermediate-high RR (*n* = 4)	*n* = 3, microscopic invasion of surgical margins
		*n* = 1, 40% of tumor dedifferentiation
	NIFTP (*n* = 1)	
Intermediate RR (*n* = 17)	Low RR (*n* = 3)	*n* = 1, classic PTC (previously PTC tall cell variant)
		*n* = 2, all LN metastasis < 0.2 cm
	Intermediate RR (*n* = 8)	
	Intermediate-high RR (*n* = 5)	*n* = 4, microscopic invasion of surgical margins (n = 3 of them with LN metastasis > 3 cm)
		*n* = 1, extranodal extension in > 3 LN metastases
	NIFTP (*n* = 1)	

MRSS ATA 2009: modified risk stratification system from ATA 2009 guidelines; RR: risk of recurrence; NIFTP: noninvasive follicular thyroid neoplasm with papillary-like nuclear features; HPS: historic pathology slide; PTC: papillary thyroid carcinoma; LN: lymph node.

Out of 17 patients initially classified as intermediate RR, 3 were reassigned to the low RR group (1 because of a change in the histology variant and 2 due to the presence of micrometastasis in all involved lymph nodes) whereas 5 patients were reclassified to the intermediate-high category (4 because of surgical margins involved and 1 because more than 3 metastatic lymph nodes with extranodal extension were present). Three patients with positive surgical margins also had at least 1 lymph node metastasis > 3 cm in the largest dimension. One patient who initially was assigned to the intermediate RR group was diagnosed as NIFTP (Table 4).

Finally, the percentages of initial response to therapy and the final clinical outcomes were compared between the initial RR assessed before and after the pathology slide review. The percentage of SIR at initial evaluation changed from 6.7 to 2.4% (*p* = 0.6254) in the low risk group and from 12.5 to 20% (*p* = 0.6171) in the intermediate risk group (Table 5). The frequency of SIR at final follow-up changed from 2.2 to 0% (*p* = 1) in patients with low RR and from 6.3 to 20% (*p* = 0.5304) in patients with intermediate RR (Table 6).

**Table 5:** Comparison of the initial response to therapy to risk of recurrence assessed before and after HPS review

		Initial response to therapy (*n* = 61)^a^			
		Excellent	Indeterminate	Biochemical incomplete	Structural incomplete
MRSS ATA 2009					
	Low RR (*n* = 45)	27 (60%)	14 (31.1%)	1 (2.2%)	3 (6.7%)
	Intermediate RR (*n* = 16)	6 (37.5%)	4 (25%)	4 (25%)	2 (12.5%)
MRSS ATA 2009 after HPS review^a^					
	Low RR (*n* = 42)	30 (71.4%)^b^	10 (23.8%)	1 (2.4%)	1 (2.4%)^b^
	Intermediate RR (*n* = 10)	4 (40%)^c^	0 (0%)	4 (40%)	2 (20%)^c^
	Intermediate-high RR (*n* = 9)	3 (33.3%)	1 (11.1%)	3 (33.3%)	2 (22.3%)

MRSS ATA 2009: modified risk stratification system from ATA 2009 guidelines; RR: risk of recurrence; NIFPT: noninvasive follicular thyroid neoplasm with papillary-like nuclear features; HPS: historic pathology slide.

a *n* = 2 patients reclassified as NIFTP were excluded from the analysis. They both had an initial excellent response.

b Comparison between risk of recurrence classification before and after historic pathology slide review in low risk patients: excellent response (60% *vs*. 71.4%, *p* = 0.3668), structural incomplete response (6.7% *vs*. 2.4%, *p* = 0.6171).

c Comparison between risk of recurrence classification before and after historic pathology slide review in intermediate risk patients: excellent response (37.5% *vs*. 40%, *p* = 1), structural incomplete response (12.5% *vs*. 20%, p = 0.6254).

**Table 6:** Comparison of the final response to therapy between risk of recurrence assessed before and after HPS review

		Final response to therapy^a^ (*n* = 61)			
		Excellent	Indeterminate	Biochemical incomplete	Structural incomplete
MRSS ATA 2009					
	Low RR (*n* = 45)	31 (68.9%)	12 (26.7%)	1 (2.2%)	1 (2.2%)
	Intermediate RR (*n* = 16)	7 (43.7%)	6 (37.5%)	2 (12.5%)	1 (6.3%)
MRSS ATA 2009 after HPS review^a^					
	Low RR (*n* = 42)	31 (73.8%)^b^	10 (23.8%)	1 (2.4%)	0 (0%)^b^
	Intermediate RR (*n* = 10)	3 (30%)^c^	3 (30%)	2 (20%)	2 (20%)^c^
	Intermediate-high RR (*n* = 9)	3 (33.33%)	3 (33.33%)	3 (33.33%)	0 (0%)

MRSS ATA 2009: modified risk stratification system from ATA 2009 guidelines; RR: risk of recurrence; NIFTP: noninvasive follicular thyroid neoplasm with papillary-like nuclear features; HPS: historic pathology slide.

Follow-up of the entire cohort (months): mean 42.6 ± 3.3, median 39.3 (range 36.1-67.9).

a 2 patients reclassified as NIFPT were excluded from the analysis. They had excellent response at final outcome (36 and 38 months of follow-up).

b Comparison between risk of recurrence classification before and after historic pathology slide review in low risk patients: excellent response (68.9% *vs*. 73.8%, *p* = 0.6431), structural incomplete response (2.2% *vs*. 0%, *p* = 1).

c Comparison between risk of recurrence classification before and after historic pathology slide review in intermediate risk patients: excellent response (43.5% *vs*. 30%, *p* = 0.6913), structural incomplete response (6.3% *vs*. 20%, *p* = 0.5304).

## DISCUSSION

A detailed pathology review of the historic histological samples of 63 DTC patients initially classified as low or intermediate RR by a validated risk stratification system (7–11) demonstrated that, considering specific features (extent of extranodal extension, extent of vascular invasion, and surgical margin evaluation, among others), the change in percentage of SIR at initial and final evaluation did not significantly differ from the statistical point of view. However, adding these new variables to the RR classification might confer a more accurate estimation of the probability of having an SIR at final follow-up. This frequency was 6.3% in the intermediate RR group previous to the pathologic review and 20% after adding specific histological data, which is more similar to what it was reported by larger cohorts of DTC patients (21%–34%) (7–11). Also, the excellent response at final outcome was achieved in 68.9% of low RR patients, but when patients were reclassified after the pathologic review, this percentage rose to 73.8% (without statistical difference).

Two patients from this cohort were diagnosed with the encapsulated variant of follicular thyroid carcinoma, which is currently known as NIFTP (15,16). These tumors are characterized by a follicular growth pattern without papillae formation, nuclear features of papillary thyroid carcinoma, encapsulation or clear demarcation, and no vascular or capsular invasion (15). They are associated with an indolent behavior and a very low risk of adverse outcome (15). The encapsulated forms of the follicular variant of PTC also have a distinct molecular profile, harboring predominantly *RAS* mutations in contrast with the more frequent *BRAF* mutations in the infiltrative forms (17,18). Currently, NIFTP is assumed to represent nearly two-thirds of the follicular variant PTCs (15,19,20). Although the ATA 2015 task force has recently proposed that this neoplasm with very low potential for recurrence should be reclassified as a non-cancer (16), and the 2 patients with NIFTP from our cohort had an excellent response to therapy (both treated with total thyroidectomy and radioiodine ablation), isolated cases of distant metastasis have been reported (21–23).

It is noteworthy that 4 patients initially classified as having a low RR were reassigned to the intermediatehigh RR group. Although it is difficult to draw a conclusion with this small cohort of patients (*n* = 9), this subgroup of patients showed 22% of SIR at initial evaluation, similar to what is expected in the intermediate RR group of patients (7–11). Indeed, all of them achieved an excellent response at final follow-up. Therefore, it seems that the presence of surgical margins involved in the microscopic analysis, the presence of extranodal extension in more than 3 metastatic lymph nodes, and < 50% of tumor dedifferentiation would probably have less impact on the percentage of SIR than the macroscopic invasion of tumor into the perithyroidal soft tissues or the presence of distant metastasis (1,7–10). It seems that, in the absence of extrathyroidal extension in small primary tumors, the positive margin status does not appear to be a poor prognostic indicator for local recurrence and has an excellent local recurrence-free survival, comparable to those with negative margins, at least in patients who received radioiodine remnant ablation, as it happened in our cohort (24).

The presence of vascular invasion is recognized as a variable that has a negative impact on the RR (25–27), and it should be informed in the pathological report. It is diagnosed as a direct tumor extension into the blood vessel lumen typically attached to the wall and covered by a fibrin thrombus (27). Minor vascular invasion (small number of foci confined to intracapsular vessels) appears to have a low risk of SIR (less than 5%) (28–30). On the contrary, more than 4 foci of vascular invasion are associated with a poor prognosis, mainly in follicular carcinomas (30–32). This situation seems to be uncommon in papillary carcinomas, even more in micro PTC, with a prevalence of less than 4% (25,26,28,33–35). In fact, none of the 63 patients from our cohort had vascular invasion in more than 4 foci in the pathological review.

In our cohort, we considered the oncocytic variant of PTC as an aggressive histology such as columnar cell and tall cell subtypes. The oncocytic change in PTC patients was reported to have a higher recurrence rate than PTC patients without oncocytic change (30.8% *vs*. 11.7%) (36).

One limitation of this study is the low number of patients in each RR group, which could have limited the statistical analysis. Given 2.2% and 0% SIR in the low risk groups and 6.3 and 20% SIR in the intermediate risk groups, a type I error of 5% (two-sided), and an interval confidence of 95%, a total of 966 low risk patients and 218 intermediate risk patients would have to be included to achieve a statistical power of at least 80%. It is necessary to validate these results in larger cohorts of patients. Another limitation is that every single patient in our cohort, even those classified as low risk, was treated with total thyroidectomy and RAI, a common practice in the past in most countries of Latin America (37). Currently, this practice of care may not apply to many institutions where low risk patients do not receive remnant ablation, including in our hospital (14). Therefore, further analysis is necessary to validate our results in low risk patients who do not receive RAI.

In conclusion, a detailed report of specific features in the HPR of patients with DTC might give a more accurate RR classification and a better estimation of the response to treatment. If these data are not available, the RR can also be estimated, but the expected SIR might be slightly different, although not statistically significant, as we could see in our analysis.
